# Arginine vasopressin and copeptin: comparative review and perspective in veterinary medicine

**DOI:** 10.3389/fvets.2025.1528008

**Published:** 2025-03-14

**Authors:** Mathieu Victor Paulin, Thomas Schermerhorn, Suraj Unniappan, Elisabeth C. R. Snead

**Affiliations:** ^1^Department of Small Animal Clinical Sciences, Western College of Veterinary Medicine, University of Saskatchewan, Saskatoon, SK, Canada; ^2^Department of Clinical Sciences, College of Veterinary Medicine, Kansas State University, Manhattan, KS, United States; ^3^Department of Veterinary Biomedical Sciences, Western College of Veterinary Medicine, University of Saskatchewan, Saskatoon, SK, Canada

**Keywords:** ADH, AVP, desmopressin, water metabolism, PUPD

## Abstract

Although arginine vasopressin (AVP) deficiency, AVP resistance, and primary polydipsia are important causes of polyuria and polydipsia (PUPD), measurement of AVP has never been implemented as a routine diagnostic test for patient care in either human or veterinary medicine, due to significant challenges with the methodologic reliability of laboratory assays for measuring AVP. Responses to a modified water deprivation test and/or a desmopressin acetate trial have been used as indirect markers of AVP deficiency or resistance. However, interpretations of these tests can be especially challenging in cases of partial AVP deficiency or resistance. Over the past decade, plasma copeptin (CoP), a glycopeptide comprising the C-terminal part of the AVP preprohormone, has mostly replaced AVP measurement in humans. When combined with CoP-based stimulation tests, such as hypertonic saline and arginine stimulation tests, plasma CoP measurement offers excellent diagnostic accuracy for the diagnosis and differentiation of cases of central diabetes insipidus (DI), nephrogenic DI, and primary polydipsia in humans. In dogs, CoP has recently been measured in saliva and serum using canine or human enzyme-linked immunosorbent assays. This review will provide an update on the physiologic regulation of AVP production and secretion, the limitations of its measurement in human and veterinary medicine, as well as a summary of the indications and performance of CoP measurement in human and veterinary medicine to date. This is with a purpose to encourage validation and implementation of CoP measurement in veterinary medicine.

## 1 Introduction

Arginine vasopressin (AVP), also referred to as anti-diuretic hormone (ADH), plays a key role in water metabolism. Despite the central role of AVP in the pathophysiology of numerous diseases, measurement of AVP has never been implemented as a routine diagnostic test for patient care in either human or veterinary medicine due to limitations and concerns regarding the methodologic reliability of current laboratory assays (1, 2). This is illustrated best in humans with polyuria-polydipsia (PUPD) disorders, where plasma AVP measurements have seldom been performed and instead indirect assessments of AVP secretion and action have been relied on to differentiate between PUPD disorders (1). Similarly, there is a major gap in knowledge in veterinary medicine since plasma AVP measurement is not commercially offered by laboratories.

Over the past 10 years, plasma copeptin (CoP), a 39-amino acid glycopeptide comprising the C-terminal part of the AVP preprohormone precursor that is co-secreted with AVP, has become a surrogate marker for plasma AVP in humans with PUPD disorders. It has been especially useful in distinguishing primary polydipsia (PP) from PUPD caused by different forms of diabetes insipidus (DI). Diabetes insipidus can be subclassified into two broad categories depending on whether the disorder is secondary to decreased synthesis and secretion of AVP (Central DI – CDI), or to insensitivity of the renal distal tubules and collecting ducts to AVP leading to a decreased insertion of AQP2 channels into the apical cell membrane of the renal principal cells and a subsequent decrease in AQP2–mediated free water reabsorption (Nephrogenic DI – NDI) (3). Central DI is often referred to as “AVP deficiency” while NDI is often referred to as “AVP resistance” (4). Central DI can be subclassified based on diminished (partial CDI) or absent (complete CDI) AVP secretion in response to appropriate osmotic or non-osmotic stimulation (4). Likewise, NDI can be subclassified into partial (partial NDI) or complete (complete NDI) subtypes based on the degree of resistance of the renal distal tubule and collecting ducts to AVP action. Furthermore, since its discovery, CoP has been investigated as a diagnostic and prognostic marker in many other acute and chronic disorders, including as a marker of critical illness and heart failure (5).

This review will examine the current understanding of AVP regulation in PUPD disorders, the challenges of measuring AVP in humans and small animal companions, and the potential of CoP measurement as a diagnostic tool in both fields. Our goal is to promote the development and adoption of CoP measurement in veterinary practice.

## 2 Physiology of water metabolism

### 2.1 Arginine vasopressin synthesis and secretion, major and minor regulatory pathways

**Figure 1:**
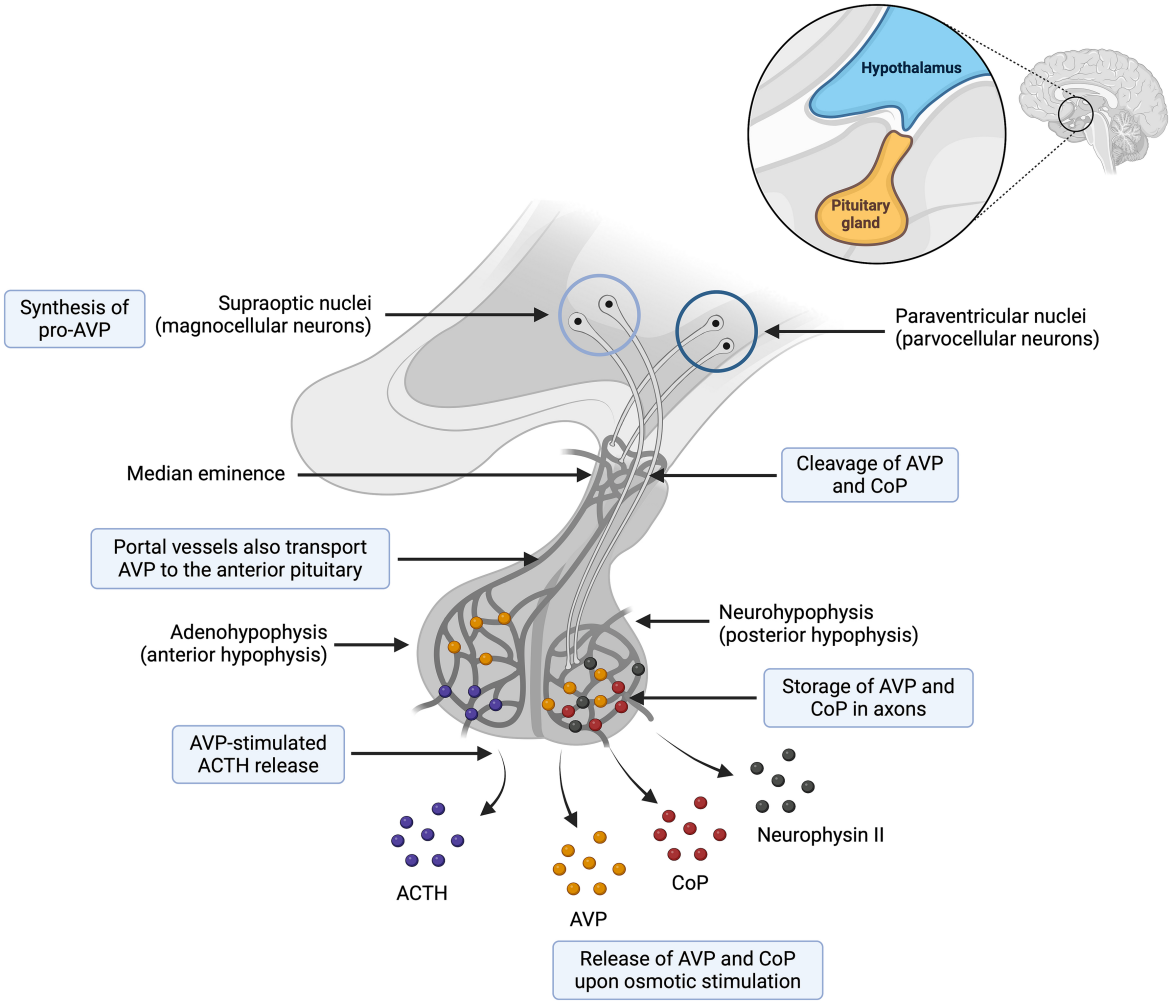
Synthesis and secretion of arginine vasopressin (AVP) and copeptin (CoP). The preprohormone provasopressin (prepro-AVP) is mainly synthesized in the magnocellular neuron and to a lesser extent in the parvocellular neurons. Pro-AVP is packaged into neurosecretory granules for axonal transport from the magnocellular neurons to the neurohypophysis. Several cleavages occur during axonal transport and lead to three main end products (AVP, CoP and neurophysin II), which are secreted in equimolar amounts. To a lesser extent, an accessory neurosecretory pathway transports AVP from hypothalamic parvocellular neurons to the adenohypophysis, where AVP acts synergistically with corticotropin-releasing hormone (CRH) to stimulate the release of adrenocorticotropic hormone (ACTH). Adapted from Christ-Crain et al. (57). Created with BioRender.com (2024).

Arginine vasopressin is the antidiuretic hormone in all mammals except swine and other members of the suborder Suina (in which lysine in lieu of arginine vasopressin is synthesized) (6). Its synthesis and secretion depend on a major and a minor pathway (Figure 1). The major pathway (also referred to as the osmotic pathway) occurs in response to changes in plasma osmolality (pOsm), and is the more sensitive regulatory mechanism of AVP synthesis and secretion (7). Osmolality refers to the milliosmoles of solute per kilogram of solution (8), and can be measured by freezing point depression or estimated *via* several equations (Table 1) (8, 9). Plasma sodium is the principal determinant of pOsm. Baseline measured pOsms range from 290 to 310 mOsm/kg in healthy dogs and 290–330 mOsm/kg in healthy cats (6). A small increase in pOsm (as little as 1–2%) stimulates hypothalamic osmoreceptors located in the subfornical organ (SFO) and the osmoreceptor nuclei of the organum-vasculosum of the lamina terminalis (OVLT). Both the SFO and the OVLT belong to the thirst center and reside in the wall of the third ventricle. Because the blood brain barrier is incomplete here and there are fenestrated capillaries around the OVLT, these osmoreceptors are influenced by the osmolality of both the plasma and the cerebrospinal fluid (10). The magnocellular neurons of the supraoptic nucleus and the parvocellular neurons of the paraventricular nuclei receive information from the thirst center (10). Additionally, magnocellular neurons can be directly stimulated by changes in plasma sodium concentrations (11).

**Table 1:** Commonly used equations to estimate plasma osmolality (pOsm).

International system of units	pOsm = 2 ([Na]_plasma_ (mmol/L) + [K]_plasma_ (mmol/L)) + [glucose]_plasma_ (mmol/L) + [BUN]_plasma_ (mmol/L)
Conventional units	pOsm = 2 ([Na]_plasma_ (mmol/L) + [K]_plasma_ (mmol/L)) + [glucose]_plasma_/18 (mg/dL) + [BUN]_plasma_/2.8 (mg/dL).

The minor regulatory pathway (also referred to as the non-osmotic pathway) occurs in response to changes in systemic blood pressure, intravascular volume and non-specific somatic stress (e.g., stress, sepsis, pain, hypoglycemia, exercise, vomiting, nausea). This is a less sensitive regulatory pathway since systemic blood pressure and intravascular volume are primarily regulated by the renin-angiotensin-aldosterone system (RAAS) (12). Changes in intravascular volume or systemic blood pressure are perceived by baroreceptors in the aortic arch, carotid sinus, and left atrium, and relayed to the hypothalamus *via* cranial nerves IX (glossopharyngeal) and X (vagus) (13). Decreases in systemic blood pressure, intravascular volume or increases in non-specific somatic stress produce an increase in plasma AVP concentration (14).

One should note that the terms “major” and “minor” pathways only refer to the sensitivity of the regulatory pathways to detect changes in pOsm (major), or systemic blood pressure and intravascular volume (minor). This does not correlate to the efficacy of each pathway to regulate water balance. On a daily basis, a healthy patient does not experience pronounced changes in intravascular volume and blood pressure, and AVP is mostly regulated by pOsm. However, in patients with severe hypovolemia or hypotension, the minor pathway will take precedence over the major pathway even if pOsm is normal or low (e.g., in patients with hypoadrenocortical crisis) (15).

**Figure 2:**
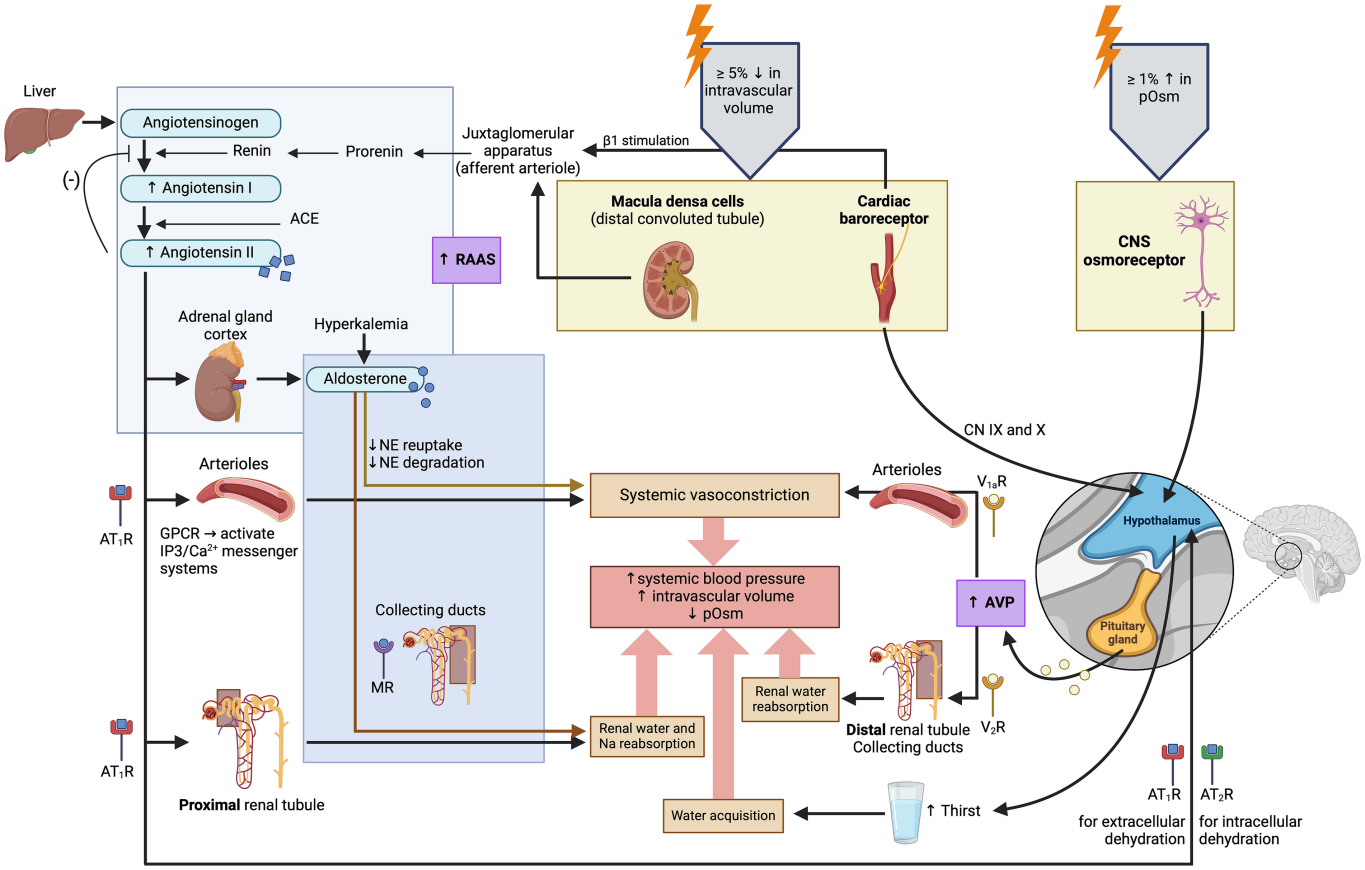
Illustration of the physiologic regulation of arginine vasopressin (AVP) synthesis and secretion. Arginine vasopressin synthesis and secretion depend on two systems: the major system involves changes in plasma osmolality (pOsm), and the minor system involves changes in systemic blood pressure and intravascular volume (7, 146). ACE, angiotensin-converting enzyme; AT_1_R, Angiotensin II receptor type 1; AT_2_R, Angiotensin II receptor type 2; CN, cranial nerve; CNS, central nervous system; GPCR, G-protein coupled receptor; MR, mineralocorticoid receptor; NE, norepinephrine; V_1a_R, vasopressin receptor 1a; V_2_R, vasopressin receptor 2; (−): inhibits. Created with BioRender.com (2024).

Arginine vasopressin synthesis and secretion involve both the hypothalamus and the neurohypophysis (also referred to as posterior pituitary) (16). As a summary, AVP synthesis occurs primarily in the magnocellular neurons of the supraoptic nucleus and, to a lesser extent, in the parvocellular neurons of the paraventricular nuclei. The axonal processes of the magnocellular neurons descend through the supraoptic hypophysial tract to the neurohypophysis, from where AVP is secreted into the bloodstream (Figure 2) (16).

More specifically, the osmotic and non-osmotic pathways stimulate synthesis of the preprohormone provasopressin (prepro-AVP) in the magnocellular neurons (1, 5, 17). Pro-AVP is packaged into neurosecretory granules for axonal transport from the magnocellular neurons to the neurohypophysis (18). During this axonal transport, several cleavages of pro-AVP lead to three main end products: AVP, CoP and neurophysin II (18). A first cleavage splits off AVP, and a second cleavage separates neurophysin II from CoP (18). When afferent stimulation depolarizes these AVP-containing neurons, the three end products (AVP, CoP and neurophysin II) are released *via* calcium-dependent exocytosis into the capillaries of the neurohypophysis in equimolar amounts (1, 2, 19). Furthermore, AVP can be synthesized but not secreted through an accessory neurosecretory pathway. This involves production of AVP by the hypothalamic parvocellular neurons and its axonal transport to the adenohypophysis, where AVP acts synergistically with corticotropin-releasing hormone (CRH) to stimulate the release of adrenocorticotropic hormone (ACTH) (20, 21). This neurosecretory pathway is stimulated by non-specific somatic stress (e.g., stress, sepsis, pain, hypoglycemia, exercise, vomiting, nausea) and constitutes a backup system for releasing ACTH without involving AVP secretion into the systemic circulation (17, 20, 21).

In humans, there is controversy about whether pulsatile AVP secretion occurs under basal conditions. In healthy dogs, basal pulsatile secretion has been shown to occur (22). Once secreted into the systemic circulation, more than 90% of AVP is bound to platelets (23), and its *in vivo* half-life is very short (less than 30 min in humans and less than 6 min in dogs) (24–26). This short half-life allows for rapid changes in circulating AVP concentration, in response to osmotic and non-osmotic pathways regulating its release; unfortunately, this significantly hampers the ability to detect and accurately measure plasma AVP concentrations (25). AVP is ultimately inactivated by endopeptidases, mainly located in plasma, the kidneys and the liver. Intact AVP and its metabolites undergo renal clearance (2).

### 2.2 Peripheral effects of arginine vasopressin

Arginine vasopressin plays a key role in total body fluid balance and therefore in maintaining vascular tone, which has led to it being referred to as a “stress hormone” (1, 2, 27). Its peripheral effects are exerted through three different receptors: V_1a_, V_1b_ and V_2_.

The V_1a_ receptor is a phosphatidylinositol-dependent receptor expressed on vascular smooth muscle cells and renal juxtaglomerular cells, leading to systemic arteriolar vasoconstriction and inhibition of renin synthesis and secretion, respectively (28).

The V_1b_ receptor (also referred as “V_3_ receptors”) is expressed by pituitary corticotrophs (29), and leads to AVP-stimulated ACTH release in the anterior pituitary (**paragraph 2.1**) (1, 30).

**Figure 3:**
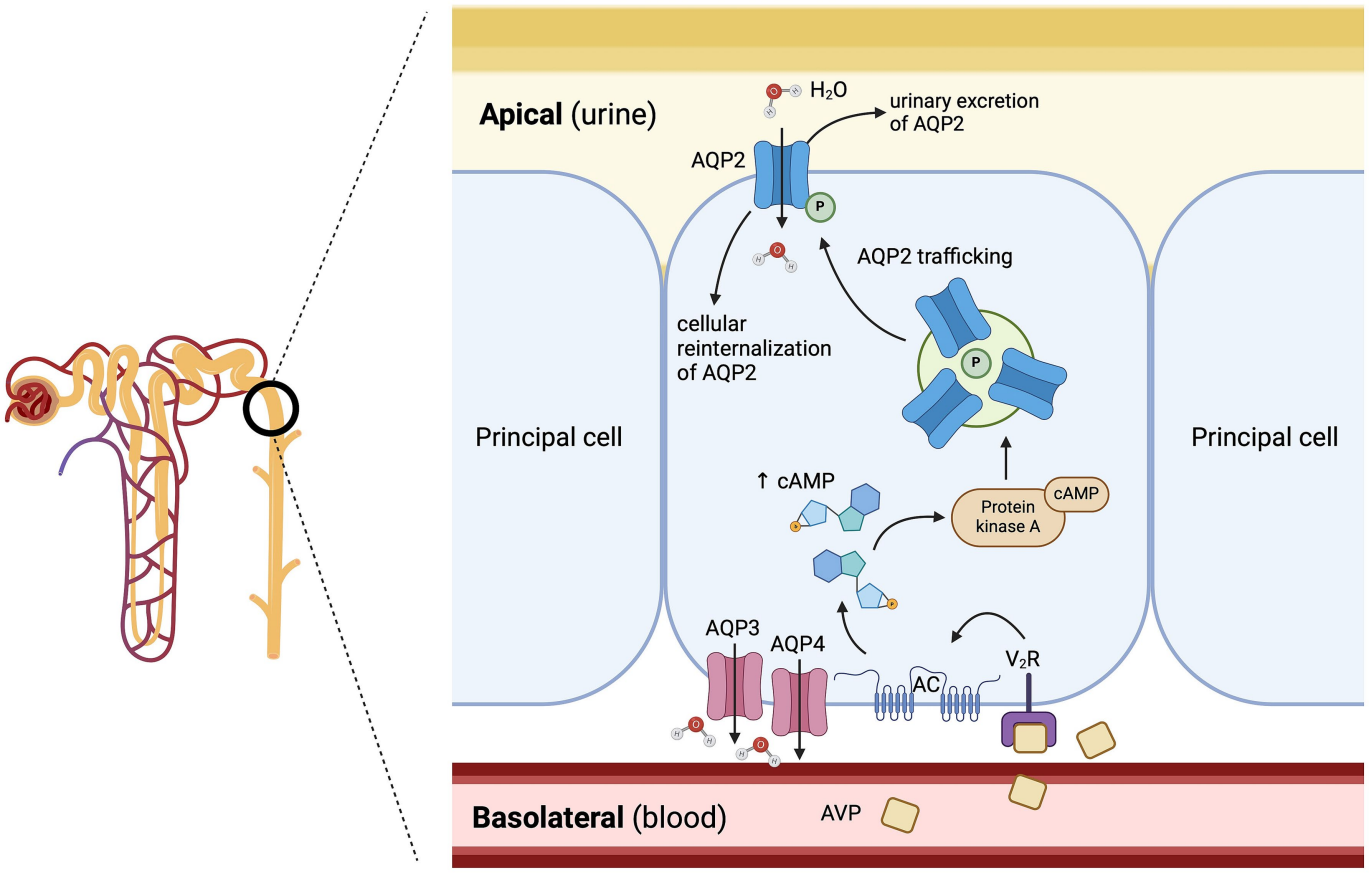
Mechanisms of free water reabsorption *via* the aquaporin-2 (AQP2) channels in the renal distal tubule and collecting duct. Arginine vasopressin (AVP) binds to cAMP-dependent-V_2_ receptors on the basolateral surface of the principal cells in the distal nephron, leading to the activation of a G-protein pathway, which stimulates the insertion of aquaporin-2 channels (AQP2) into the apical cell membrane. The osmotic gradient between the hypertonic medullary interstitium and the hypotonic filtrate in the distal nephron allows the passive movement of free water through the AQP2 channels. AC: adenylyl cyclase; cAMP: cyclic adenosine monophosphate; P: phosphorylated; V_2_R: arginine vasopressin receptor 2. Adapted from Kavanagh et al. (34). Created with BioRender.com (2025).

The V_2_ receptor is a G-protein-coupled receptor (GPCR) mainly expressed in the renal distal tubule and collecting ducts, and plays a key role in renal water reabsorption (28). As a brief reminder, approximately two thirds of the filtered water is passively reabsorbed, following the active reabsorption of, for example, sodium and glucose, in the renal proximal tubules (31). As the sodium is selectively reabsorbed without free water in the ascending loop of Henle, notably by the Na^+^-K^+^-2Cl^−^ cotransporter in the thick ascending limb, this results in a hypotonic urine entering the distal tubule (32). Approximately 90% of the remaining water is then reabsorbed as free water in the renal distal tubule and collecting ducts under the influence of AVP (6, 33). More specifically, AVP binds to cAMP-dependant-V_2_ receptors (V_2_R) on the basolateral surface of the principal cells in the distal nephron, which activates the Gs adenylyl cyclase system. The activation of the G-protein pathway increases intracellular levels of cyclic 3′,5′-adenosine monophosphate, which activates protein kinase A, and subsequently leads to phosphorylation of preformed aquaporin-2 (AQP2) water channels localized in intracellular vesicles followed by their insertion into the apical cell membrane (Figure 3) (34). The osmotic gradient between the hypertonic medullary interstitium (generated by the countercurrent multiplier system of the nephron) and the hypotonic filtrate in the distal nephron allows the passive movement of free water through different AQP channels: first, from the distal tubule into the cytoplasm of principal cells (mediated by AQP2 located at the apical pole of the principal cells), and secondly from the cytoplasm of principal cells to the renal medulla (mediated by AQP3 and AQP4 located at the basolateral pole of the principal cells) (34). The vasa recta renis reabsorbs this free water from the renal medulla back into the systemic circulation, which helps maintain the persistent osmotic gradient between the hypertonic medullary interstitium and the hypotonic filtrate (1, 6, 30, 34). The synergistic mechanism of free water renal reabsorption *via* the interaction of AVP with V_2_ receptors, and systemic arteriolar vasoconstriction *via* its interaction with V_1a_ receptors, is crucial to maintaining tissue perfusion, especially in case of hypovolemia and systemic hypotension (1, 30). Dissociation of AVP from the V_2_ receptor leads to decreased intracellular cAMP concentration and subsequent cellular reinternalization of the AQP2 channels; the distal tubule and collecting duct becomes impermeable to water again (28). In conditions of plasma hypotonicity where AVP synthesis and secretion are suppressed, the urine osmolality (uOsm) may be as dilute as 20 mOsm/kg, whereas uOsm measurement above 2000 mOsm/kg has been reported in conditions of plasma hypertonicity where AVP secretion is maximal (6).

Additionally, since AQP2 channels are partially excreted in the urine, the urinary AQP2 excretion rate measurement has been investigated in humans and in dogs as a potential marker of collecting-duct responsiveness to AVP (35). In humans, urinary AQP2 excretion closely parallels changes in AVP action, with urinary AQP2 excretion decreasing under plasma hypoosmolar conditions and increasing under plasma hyperosmolar conditions or administration of an AVP analog (36–40). In dogs, AQP2 urinary excretion rate has also been shown to closely correlate with changes in AVP after water loading, hypertonic saline infusion and intravenous administration of desmopressin acetate (DDAVP) (35). Despite these initial promising results, routine measurement of AQP2 urinary excretion rate is not readily available commercially.

As a side note, V_2_ receptors are also expressed on vascular endothelium, and binding of AVP to these endothelial receptors induces the release of coagulation factor VIII and von Willebrand factor (vWF) from Weibel Palade bodies (6, 41). The pathophysiology of V_2_ receptors on vascular endothelium is beyond the scope of this review article, but explains why synthetic analogs of AVP can be administered to patients with Type I Von Willebrand’s disease to promote release of both factor VIII and vWF, or given to canine blood donors prior to blood collection to produce cryoprecipitate.

### 2.3 Desmopressin acetate

**Figure 4:**
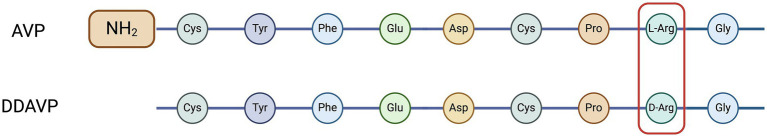
Amino acid sequences of arginine vasopressin (AVP) and desmopressin acetate (DDAVP). Adapted from Nelson (6). Created with BioRender.com (2024).

Desmopressin acetate (DDAVP) is a commercially available synthetic form of AVP with an increased antidiuretic effect to pressor effect ratio. Enhanced antidiuretic activity is obtained by substituting l-arginine with d-arginine at amino acid position 8 (Figure 4) (42). This change confers higher affinity for V_2_ receptors and lower affinity for V_1a_ receptors, and a longer half-life compared to endogenous AVP (50 min vs. < 6 min when administered intravenously) (24–26, 43). Desmopressin acetate is available in multiple formulations for oral, subcutaneous, intravenous, intra-nasal and ophthalmic administration, and is commonly used for treating CDI, or Type I Von Willebrand’s disease (2, 44).

## 3 Copeptin

**Figure 5:**
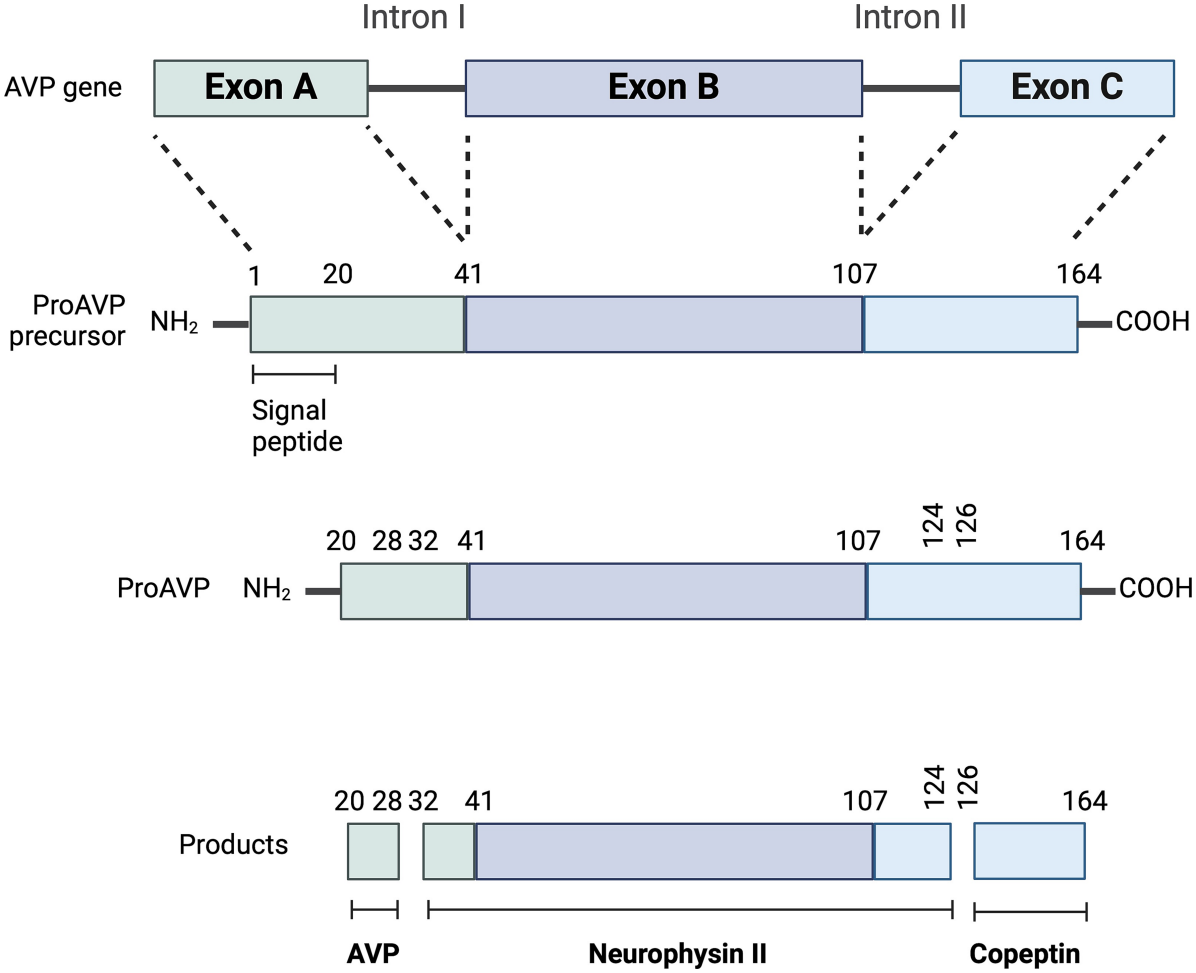
Synthesis of copeptin (CoP). Enzymatic cleavages of pro-arginine vasopressin (AVP) peptide generate AVP, neurophysin II and CoP. Adapted from Christ-Crain et al., (2) and Christ-Crain et al. (57). Created with BioRender.com (2024).

Copeptin is one of the three main end products of cleavage of the prepro-AVP hormone, along with AVP and neurophysin II (Figure 2). All three products are released into the systemic circulation in equimolar amounts (2, 5, 45). Copeptin was first isolated in pigs in 1972 (46), and is a 39-amino acid glycopeptide that comprises the C-terminal part of prepro-AVP (Figure 5) (1, 2, 5). For this reason, CoP has also been referred to as AVP-associated glycopeptide (1, 2).

The physiologic role of CoP remains unknown (1, 2). Early experiments suggested a role as a prolactin-releasing factor (47, 48). More recent data showed that CoP interacts with the calnexin-calreticulin system, which monitors protein folding and interacts with glycosylated proteins (49, 50). Therefore, CoP may be a chaperone-like molecule involved in the structural formation of pro-AVP (2, 51). Whether the absence of CoP and subsequent inefficient protein folding and maturation of pro-AVP has a role in the pathogenesis of CDI remains to be determined (2).

The catabolism of CoP has not been thoroughly investigated yet (2), but its measurement in the urine indicates at least partial renal excretion (52). In humans with chronic kidney disease, plasma CoP concentration is inversely correlated with decreasing glomerular filtration rate, and positively correlated to albuminuria or proteinuria (53, 54). Furthermore, increased CoP levels have been associated with worse outcomes in chronic kidney disease and hypertension (54, 55). Studies on elimination kinetics are scarce, but one study suggested CoP half-life was approximately 2 times longer than AVP half-life in humans (56).

Copeptin can be measured in either serum or plasma. Since CoP is secreted in equimolar amounts with AVP, it has been used as a sensitive surrogate for plasma AVP secretion in humans (57, 58). In one study in humans, serum CoP concentrations correlated as expected with plasma AVP under variable plasma osmolality states: hyperosmolality following water deprivation testing or intravenous administration of hypertonic saline, and hypoosmolality following oral administration of tap water and intravenous administration of hypotonic saline (59). In a more recent study, pOsm, plasma CoP and plasma AVP concentrations were measured in human volunteers at baseline, after an oral water load, and during and after intravenous infusion of 3% hypertonic saline, and a close correlation between plasma AVP and plasma CoP concentrations was found (Spearman’s rank correlation coefficient = 0.80) (58). Interestingly, this study also showed a stronger correlation between pOsm and CoP (*r* = 0.77) than between pOsm and AVP (*r* = 0.49) (58).

In humans, copeptin measurements can be reliably interpreted independently of the patient’s age (60), the time point of sampling (61, 62), food intake (63), and any influence from reproductive hormones during the menstrual cycle (64). Men were found to having slightly higher plasma CoP values than women in multiple studies (2, 60, 65).

## 4 Laboratory measurement of plasma arginine vasopressin and copeptin

### 4.1 Measurement of plasma arginine vasopressin and copeptin in human medicine

There are serious issues regarding the methodological reliability of laboratory assays for plasma AVP measurement in humans (1, 2), and to the authors’ knowledge, there is no commercial laboratory in North America offering AVP measurement in veterinary medicine. Hence, despite the crucial physiologic role of AVP in water metabolism and vascular tone, routine measurement of AVP has never been implemented as a routine diagnostic test in either human or veterinary medicine (1).

Because of its small size, AVP cannot be measured by a sandwich immunoassay but only by less sensitive competitive immunoassays requiring a large volume (≃ 1 mL) of plasma (1, 5). AVP also has a short half-life (< 30 min) and is very labile in isolated plasma, even when stored at −20°C (5, 66). The use of protease inhibitors during collection is fundamental, and the sample needs to be kept on ice and rapidly centrifuged with the harvested plasma immediately frozen at −80°C (1, 5). Most competitive AVP immunoassays have a turnaround ≥12–24 h due to the extensive incubation steps involved in the assays and the need for an extraction step (1, 5). The low sensitivity of the assay often leads to non-detectable plasma AVP concentrations in patients with low or low-normal osmolality (5). This is partly due to the fact that ≥90% of circulating AVP is bound to platelets (23). Conversely, incomplete platelet removal from plasma samples and thrombocytosis can lead to falsely increased plasma AVP concentrations (23, 67).

Over the past decade, CoP had replaced the measurement of AVP in human medicine for the investigation of polyuria-polydipsia disorders due to its many advantages (5). Unlike AVP, CoP is extremely stable in plasma or serum *ex-vivo* and the use of protease inhibitors in the tube of collection is not required (1). *Ex-vivo* CoP stability, as defined by <20% loss of recovery, is ≥7 days in both plasma or serum when stored at room temperature and ≥ 14 days at 4°C (60). Plasma or serum CoP are routinely measured using the original sandwich immunoluminometric assay (LIA) (60), or its automated immunofluorescent successor on the KRYPTOR platform (2). Both assays require a small amount of plasma (≃ 50 μL) and have a short turnaround of 0.5–2.5 h since they do not necessitate an extraction step (2). Various enzyme-linked immunosorbent assays (ELISAs) are also available for research purposes but are not recommended for human clinical studies (2). Both LIA and KRYPTOR assays have good agreement; however, in one study, CoP measured by an ELISA assay correlated poorly with both the LIA and KRYPTOR assays (68). Moreover, the current cut-off values for plasma CoP that are used to investigate PUPD disorders have been developed and validated using the LIA and KRYPTOR assays only (68).

### 4.2 Measurement of plasma arginine vasopressin and copeptin in veterinary medicine

Plasma AVP has seldom been reported in veterinary medicine, especially in the last 20 years, likely due to how cumbersome the current ELISA assays are and hence the lack of a commercially available assay. Multi-species AVP radioimmunoassay kits (69, 70), human AVP Enzyme Immune Assay (EIA) (71) and canine AVP ELISAs (72) have been used for measuring AVP in dogs. To the authors’ knowledge, no AVP assay is commercially available in North America for veterinary clinical use.

The current literature reporting on the measurement of CoP in small animal medicine includes a single research paper (73), a case report (74), and three abstracts (75–77), and only in the canine species. In the single paper (2022), the authors measured salivary CoP in dogs diagnosed with separation-anxiety related problems that were subjected to a short separation test (73). Salivary CoP was measured using a commercially available ELISA kit from BlueGene Biotech (Shanghai, China) designed for the quantitative determination of salivary CoP in dogs. The mean CoP recovery was 98.5% ± 6.3, the average intra- and inter-assay coefficient of variation (CV) were 4.5–5.9% and 6.8–8.2%, respectively, and the assay sensitivity was 1 pg./mL The case report described the long-term outcome of a chihuahua with idiopathic syndrome of inappropriate antidiuretic hormone secretion, and reported serum CoP measurement using the canine copeptin ELISA Kit from MyBioSource (San Diego, United States) (74). In this case report, analytical performances of the ELISA assay were not reported. The first abstract (2019) validated a canine copeptin ELISA Kit (MyBioSource, San Diego, US) in diabetic and non-diabetic dogs (76). The limit of detection was 50 pg./mL with an inter-assay CV at 12% and an intra-assay CV at 14%; linearity was determined by diluting samples into 1:2 and 1:4 dilution (76). The second abstract (2023) validated a human copeptin ELISA Kit (Phoenix Pharmaceutical Inc., Burlingame, US) in healthy dogs participating in a voluntary blood donor program (75). Intra-assay CV was 8.42% and inter-assay CV was 9.6%. The limits and range of detection were 0.1–10 ng/mL. The assay displayed dilutional linearity and parallelism. Recovery from spiked samples averaged 147% (75).The third and most recent abstract (2024) reported the measurement of plasma AVP and serum CoP concentrations under hypo-, iso- and hyper-osmolar conditions in dogs, using the canine copeptin ELISA Kit from MyBioSource (San Diego, United States) (77). Analytical performances of the ELISA assay were not reported in the abstract, although a mean intra-assay and inter-assay CV of 3.5% and 6.0%, respectively, were reported during the oral presentation at the American College of Veterinary Medicine Forum 2024.

## 5 Diagnostic challenges of diabetes insipidus and primary polydipsia in dogs and cats

### 5.1 Diabetes insipidus and primary polydipsia in dogs and cats

Polyuria-polydipsia is a common reason for patient presentation in small animal veterinary medicine. Polydipsia is defined by a water consumption >100 mL/kg/24 h in dogs, and > 45 mL/kg/24 h in cats (6). Polyuria is defined by a urine production >50 mL/kg/24 h (or > 2 mL/kg/h) in both dogs and cats (6). The complete approach to the differential diagnosis of PUPD is beyond the scope of this review, but common causes of PUPD include chronic renal disease, hyperadrenocorticism, diabetes mellitus, hyperthyroidism, post-obstructive diuresis, hypercalcemia, liver disease, hypoadrenocorticism, hypokalemia, pyelonephritis, pyometra, salt administration, and intravenous fluid administration (6). Most etiologies can be diagnosed by routine blood work, urinalysis and routine imaging examinations (e.g., chest radiographs, abdominal ultrasound). Once common causes of PUPD have been ruled out, clinicians are often left with PP and DI (6, 17). Diabetes insipidus results from either a partial or complete deficiency of circulating plasmatic AVP (CDI), or from partial or complete resistance of the distal nephron to AVP (NDI). Both disorders result in increased urinary free water excretion (aquaresis) and compensatory polydipsia to minimize dehydration. Patients with complete DI produce hyposthenuric urine, while patients with partial DI can produce either hyposthenuric or isosthenuric urine (78).

Central DI is caused by a lesion at one or more sites involved in the synthesis and secretion of AVP (hypothalamic osmoreceptors, supraoptic or paraventricular nuclei, hypophyseal tract, and neurohypophysis) (1, 79, 80). In humans, CDI has been classified into 3 main categories: acquired, congenital, and gestational DI. Gestational DI in pregnant women is caused by accelerated AVP breakdown by the placental enzyme vasopressinase (62, 81–83). A similar classification has been used in veterinary medicine, but to the authors’ knowledge, gestational DI has never been reported in dogs or cats. Reported causes of CDI in dogs and cats include primary or metastatic central nervous system neoplasia (involving the hypothalamus or neurohypophysis), traumatic brain injury (the hypothalamus, hypophysis and pituitary stalk are especially vulnerable to injury), iatrogenic (e.g., hypophysectomy), congenital defects (e.g., hypoplasia, cysts, idiopathic), immune-mediated hypophysitis, infectious encephalitis, and idiopathic (6, 51, 84–90).

In nephrogenic DI, the insensitivity of the renal distal tubules and collecting ducts to AVP leads to a decreased insertion of AQP2 channels into the apical cell membrane of the renal principal cells and a subsequent decrease in AQP2–mediated free water reabsorption (3). In human and veterinary medicine, etiologies of NDI have been classified as acquired and congenital (6, 82). In dogs and cats, reported causes of acquired NDI include hyperadrenocorticism, drugs (e.g., glucocorticoids, aminoglycosides, amphotericin B), hypercalcemia, urogenital infection with *Escherichia coli* (e.g., pyometra or pyelonephritis), hypokalemia, obstructive nephropathy, neoplasms (e.g., intestinal leiomyosarcoma, pro-opiomelanocortin-producing pituitary tumor), and possibly leptospirosis (6, 91–93). Familial NDI has been reported in 3 out of 4 male puppies of a litter of Siberian Huskies, with a suggested X-linked recessive origin (94). Clinical signs occurred by 8–12 weeks of age (94). Examination of kidney membranes from NDI-affected male Siberian Huskies showed that the inner medulla had normal V_2_R numbers, but with 10-fold lower affinity for AVP (95). The affected puppies showed positive response to high-dose of DDAVP, which corroborated the finding of a qualitative defect in V_2_R characterized by a lower binding affinity (94). Congenital NDI has seldomly been reported in other breeds (Boston Terrier, Shiba Inu, Miniature Poodle, German Shepherd dogs), but its cause remains unknown (95). To the authors’ knowledge, congenital NDI has not been reported in cats.

In humans, PP results from either a defect in the thirst center or mental illness. Distinct sets of osmoreceptors of the lamina terminalis diencephalon exist for regulating AVP release and thirst (96–99). In humans, the relationship between pOsm and activation of the thirst center is just as sensitive as between pOsm and AVP release (100, 101). The presence of distinct sets of osmoreceptors of the lamina terminalis diencephalon is notably illustrated by the fact that patients with CDI rarely develop severe or sustained hypernatremia unless they have concurrent hypodipsia, or unless access to water is restricted or denied (102, 103). Conversely, most patients with primary polyuria due to an overactive thirst center have normal AVP osmoregulation (102, 103). Although PP occurs despite intact AVP secretion and renal sensitivity to AVP (2), chronically increased free water intake will eventually lead to central AVP suppression and downregulation of AQP2 channels (104), which leads to the production of hypo- or isosthenuric urine depending on the severity of PP. This makes the distinction between PP and DI especially challenging in cases of partial PP and partial DI (2). In dogs, reported causes of PP are idiopathic PP (psychogenic or behavioral disorders leading to compulsive water intake), fever, pain, central nervous system neoplasia, encephalopathies including hepatic encephalopathy and chronic enteropathies (inflammatory bowel disease and antibiotic-responsive diarrhea) (6, 105). In cats, idiopathic PP (106) and hyperthyroidism (6) have been reported as causes of PP.

### 5.2 Limitations of the water deprivation test and desmopressin trial

In humans (1) and dogs (17, 107), modified water deprivation testing (MWDT) has been performed for decades to distinguish CDI, NDI and PP. An alternative to the MWDT for distinguishing these causes of PUPD involves a DDAVP trial, which gained in popularity given the risk associated with MWDT, especially in dogs with CDI.

The MWDT include 3 phases and was designed to determine whether endogenous AVP is released in response to dehydration and whether the kidneys respond to this stimulus. During phase 1, the water intake is carefully decreased to 60–90 mL/kg/24 h over the course of 3–4 days. If the urine specific gravity (USG) increases to >1.030 in dogs or > 1.035 in cats after phase 1, PP is diagnosed and the MWDT is discontinued. During phase 2 (referred to as the water deprivation phase), the water intake is restricted until the dog loses 3–5% of its body weight to assess the effect of dehydration. Dehydration can also be confirmed if pOsm is >320 mOsm/L in dogs, but this is rarely measured in practice given the limited access to osmometers. Patients with PP should be able to concentrate their urine at the end of phase 1 or phase 2, while patients with DI cannot. Phase 3 is conducted if the patient has reached the endpoint of phase 2, but USG is still <1.030 in dogs or < 1.035 in cats, and it aims to determine the renal responsiveness to administration of a supraphysiologic dose of exogenous AVP (desmopressin acetate, DDAVP). Contraindications for performing a MWDT include pre-existing azotemia, hypercalcemia, and lack of adequate medical monitoring during the test since severe dehydration can occur within as early as 4–6 h in patients with complete DI and can lead to hypovolemia, systemic hypotension and subsequent multiple organ dysfunction syndrome (108). In humans, the WDT has a diagnostic accuracy as low as 70% to distinguish PP from complete DI, and as low as 41% to distinguish PP from partial DI (79). To the authors’ knowledge, the diagnostic accuracy of MWDT has not been reported in large populations of dogs or cats, but would not be expected to be higher. Surprisingly, the diagnostic criteria for the WDT used in human medicine was based on data obtained from only 36 patients (79). Plasma AVP measurement in response to WDT has been proposed in humans to overcome the limitations of indirect assessment, but has been precluded by the aforementioned limitations of the AVP assays (109). Similarly, one study investigated plasma AVP response to intravenous infusion of 20% hypertonic saline for 2 h in 18 dogs with polyuria that had been present in most cases since a young age (110). Three categories could be distinguished: an exaggerated response (*n* = 3), a subnormal response (*n* = 4), and a nonlinear response with high plasma AVP concentrations unrelated to increases in pOsm (*n* = 11) (110).

A DDAVP trial is also used by many clinicians and is designed to determine the patient’s responsiveness to trial therapy. Oral DDAVP tablets or conjunctival drops of DDAVP nasal spray are administered every 8 to 12 h for 7 days, and the effect of DDAVP should not be critically evaluated until after 5–7 days of therapy (6). A decrease in the severity of PUPD and an increase in USG ≥ 50% to baseline supports the diagnosis of CDI. Patients with NDI will only show minimal improvement in their clinical signs and urine concentrating ability, although a better response may be observed with very high doses of DDAVP (95). Patients with PP may also show a minimal improvement in their clinical signs and urine concentrating ability since chronic overactivity of the thirst center depresses AVP synthesis and secretion (6, 111). Therefore, the main challenge is to differentiate between NDI and PP. Theoretically, DDAVP administration to a dog with PP can result in water intoxication and hyponatremia; however, to the authors’ knowledge, no case of clinical hyponatremia has been reported in the veterinary literature, although a clinically relevant decrease in serum sodium concentrations was recently reported in some healthy dogs receiving both prednisolone and DDAVP (111).

The detailed protocols for MWDT and DDAVP trial are beyond the scope of this review, and the readers are invited to consult reference textbooks in endocrinology and nephrology.

## 6 Clinical use of copeptin measurement

### 6.1 Utility of copeptin measurement in the differential diagnosis of polyuria-polydipsia in human medicine

The main and best-validated indication for CoP measurement in humans is for the differential diagnosis of PUPD (2) and especially to distinguish between DI and PP where plasma CoP measurement has now mostly replaced plasma AVP measurement (1, 2). The most straightforward use is for patients with NDI, since plasma CoP concentrations are consistently high (2). In one study, a random baseline plasma CoP measurement without prior water deprivation >21.4 pmol/L differentiated NDI from CDI and PP with 100% accuracy, rendering a WDT unnecessary (112). Conversely, a plasma CoP concentration < 21.4 pmol/L cannot differentiate between CDI and PP and further tests are then required (2).

Given the substantial overlap in baseline plasma CoP concentration between CDI and PP patients, the distinction of these two conditions is more challenging (2). One exception is for patients with plasma CoP concentration < 2.6 pmol/L following an overnight 8-h fluid restriction, which indicated complete CDI (sensitivity 95%, specificity 100%) in one study (78). Another exception is for patients with substantial hypernatremia at the time of testing, with one study showing that a plasma CoP concentration ≤ 4.4 pmol/L had a sensitivity of 100% and specificity of 99% for the diagnosis of CDI in hospitalized patients with severe hypernatremia (> 155 mmol/L) (113). In this study, all patients with dehydration, salt overload, and NDI, had elevated plasma CoP concentrations (113). In normonatremic patients, two recent CoP-based stimulation tests have been studied to overcome the diagnostic challenge of differentiating CDI and PP using the inconvenient WDT: a hypertonic saline stimulation test (HSST) (114), and an arginine stimulation test (AST) (115).

**Figure 6:**
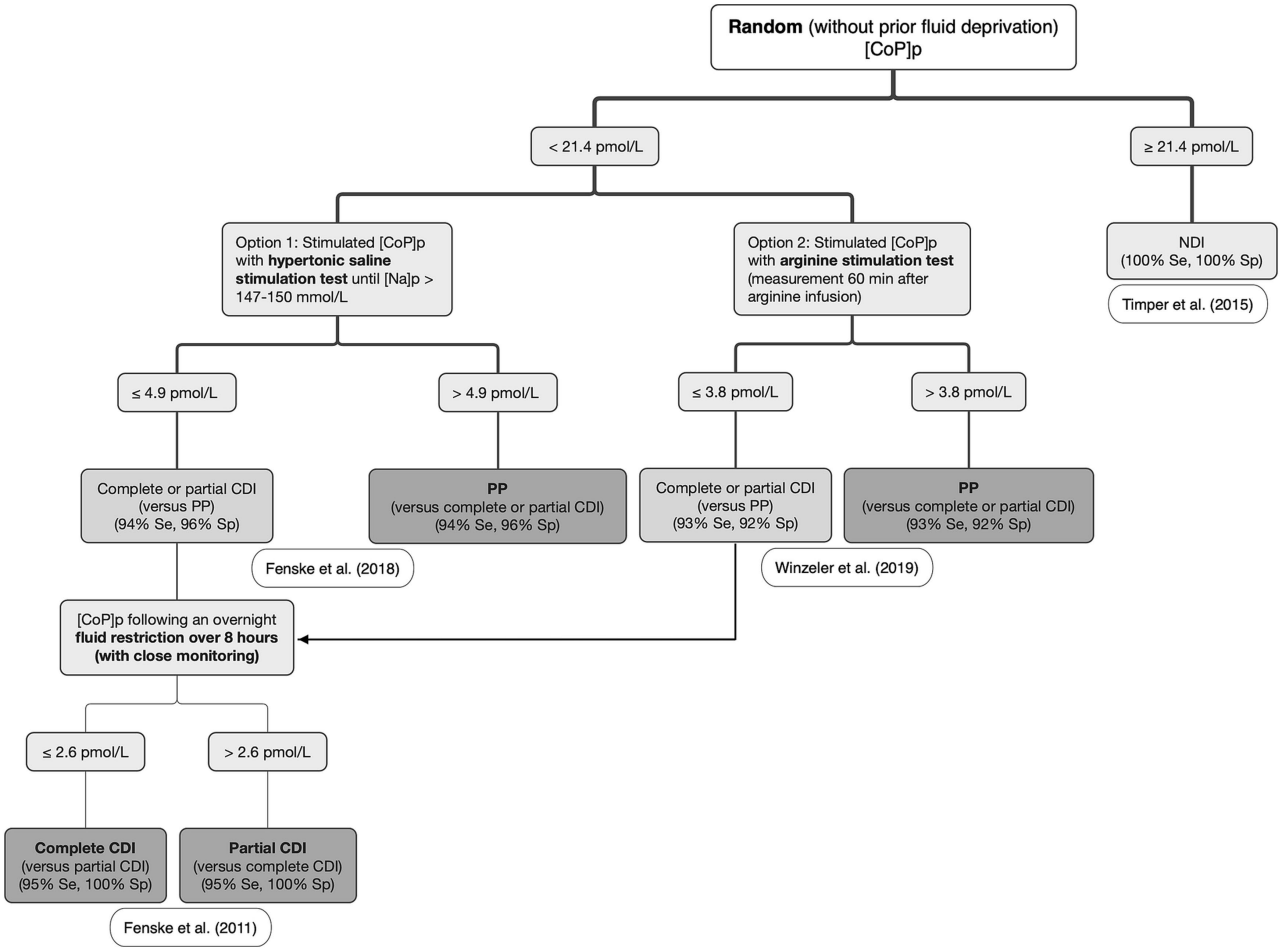
Proposed algorithm for the differential diagnosis of central diabetes insipidus (CDI), nephrogenic diabetes insipidus (NDI), and primary polydipsia (PP) in humans. [CoP]_p_: plasma copeptin concentration; [Na]_p_: plasma sodium concentration. Adapted from Christ-Crain et al., (5) and Christ-Crain et al. (57) with the results of the studies from Timper et al. (112); Fenske et al. (78); Fenske et al. (114); and Winzeler et al. (115).

In one study where plasma sodium concentration was increased to >150 mmol/L after an intravenous bolus followed by a continuous rate infusion of 3% hypertonic saline in patients with PUPD, a cut-off plasma CoP concentration of 4.9 pmol/L had a diagnostic accuracy of 97% to distinguish CDI (≤ 4.9 pmol/L) from PP (> 4.9 pmol/L) (114). The diagnostic accuracy was only slightly lower (95%) when comparing the ability to differentiate partial CDI from PP (114). The HSST was safe and well tolerated, with vertigo or headache infrequently reported (2, 114). A second option is to perform an AST. Arginine is not only a potent stimulus for the secretion of hormones from the adenohypophysis, it is also commonly used as a stimulus for growth hormone secretion when screening children with stunted growth for hyposomatotropism (116–118) and for adults with decreased growth hormone secretion secondary to traumatic brain injury (119). Arginine is also reported to be a potent stimulus for the secretion of AVP from the neurohypophysis, possibly *via* the L-arginine–nitric oxide pathway (120). One study showed that a cut-off plasma CoP concentration of 3.8 pmol/L measured 60 min after the start of intravenous arginine administration had a diagnostic accuracy of 93% to distinguish CDI (≤ 3.8 pmol/L) from PP (> 3.8 pmol/L) (115). In this study, the AST was safe and well tolerated, with the most common adverse effect being nausea (115). The slightly lower diagnostic accuracy with AST (93%) compared to HSST (97%) may be explained by the weaker stimulus of AVP secretion *via* the non-osmotic pathway. Although AST has the main advantage to not require monitoring of plasma sodium levels (115), severe nausea or vomiting are potent stimulators of AVP secretion and may decrease the test’s diagnostic accuracy (2, 115). A proposed algorithm for the differential diagnosis of CDI, NDI, and PP using plasma CoP measurement in humans can be drawn from these collective results (Figure 6). Whether a similar algorithm would be applicable in dogs and cats has yet to be determined. As CDI is uncommon, international collaborative research is encouraged, especially focusing on validating a convenient assay (as illustrated by the routine use of the KRYPTOR assay in humans) to allow the determination of serum CoP diagnostic cutoffs. Other CoP-based stimulation tests have been described including the glucagon stimulation test (121) and the insulin tolerance test, although the latter is rarely performed given the associated risk of hypoglycemia (19).

While plasma CoP measurement has tremendous value in the differential diagnosis of PUPD, its diagnostic utility is limited in the case of hyponatremia where no differentiation between patients with a diagnosis of Syndrome of Inappropriate Antidiuretic Hormone Secretion (SIADH) and other etiologies leading to hyponatremia seems possible (2, 122), with the possible exception of cerebral salt-wasting syndrome (CSWS) (1). Both SIADH and cerebral salt-wasting syndrome are characterized by hyponatremia and increased uOsm, but plasma AVP and CoP concentrations are increased with SIADH and decreased with CSWS (123–125). A recent case report described the long-term outcome of a chihuahua with idiopathic SIADH and reported a serum CoP concentration of 135.4 pg./mL, which was higher than the 75^th^ percentile for 19 healthy dogs (58.5 pg./mL) based on unpublished data from a study in Dr. Harris’s laboratory at the University of Florida mentioned in the manuscript (74). To our knowledge, CSWS has only been suspected in one cat (126), where plasma AVP concentration was not measured.

### 6.2 Utility of copeptin measurement as a biomarker of other diseases in human medicine

Although CoP measurement has mainly served as a surrogate of AVP measurement in the diagnosis of PUPD disorders, its measurement has also been reported in various diseases. A promising use of CoP measurement has been reported for the postoperative assessment of CDI following pituitary hypophysectomy (2). In one study, a plasma CoP concentration < 2.5 pmol/L on the first postoperative day following hypophysectomy had a specificity of 97% for diagnosing iatrogenic CDI, while a plasma CoP concentration > 30 pmol/L had a sensitivity of 94% to rule out iatrogenic CDI (127). In another study investigating the use of plasma CoP measurement 1 h after extubation following hypophysectomy, a plasma CoP concentration < 4.2 pmol/L indicated permanent CDI, while a plasma CoP concentration > 12.8 pmol/L excluded permanent CDI forms (128). Similarly, plasma CoP measurement may be useful in evaluating post-surgical outcome of veterinary patients after transsphenoidal hypophysectomy in dogs with pituitary-dependent hyperadrenocorticism (129), or in cats with hypersomatotropism (130, 131), albeit this has yet to be studied.

Plasma CoP measurement has also been investigated as a diagnostic and prognostic marker of septic shock, heart failure and myocardial infarction (132–134). In one study, mean plasma CoP concentration 24 h after admission to the ICU in patients with sepsis (52 ± 30 pmol/L), SIRS (88 ± 89 pmol/L) or after cardiac surgery (101 ± 82 pmol/L) was significantly greater than in 70 healthy volunteers (6 ± 3 pmol/L) (132). Another study showed that median plasma CoP concentration incrementally spiked in patients with sepsis (50.0 pmol/L), severe sepsis (73.6 pmol/L) and septic shock (171.5 pmol/L) (133). In patients with heart failure, a recent meta-analysis confirmed that elevated plasma CoP concentrations were associated with all-cause mortality, and the predictive value of plasma CoP was comparable with plasma N-terminal pro B-type natriuretic peptide (NT-proBNP) concentration for all-cause mortality (135). Increased plasma AVP may exacerbate left ventricular dysfunction by increasing systolic and diastolic wall stress (1, 136), and AVP receptor antagonist administration has therefore been suggested as a potential new treatment (137, 138).

### 6.3 Copeptin measurement in veterinary medicine

To the authors’ knowledge, the current literature reporting the measurement of copeptin in small animal medicine includes a single paper (73), one case report (74), and three abstracts (75–77), and only in the canine species. In the former, the utility of salivary CoP as a biomarker of stress was studied in dogs with separation anxiety (*n* = 13) versus dogs with no separation anxiety (*n* = 15) (73). Salivary CoP was measured in dogs before, during and after a 3-min separation from the owner in a new environment. Although salivary CoP concentration did not significantly differ between the two groups across the 3 timepoints, different CoP trends were detected in dogs with separation distress versus dogs without separation distress, and a type II error was suspected due to the small population sample size (73). The observed changes may be explained by the synergic action of AVP with CRH to stimulate the release of ACTH from the anterior hypophysis secondary to somatic stress (as outlined in **paragraph 2.1**). Similarly, and in support of this, in a study looking at plasma CoP and cortisol concentrations in human patients with varying and increasing stress levels (healthy volunteers, hospitalized patients with moderate stress, and surgical patients immediately after extubation with maximal stress), plasma CoP concentrations positively correlated with cortisol concentrations (139).

In one abstract, serum CoP concentrations in dogs with naturally occurring diabetes mellitus were higher when compared to non-diabetic dogs (76). Plasma CoP concentrations have also been reported in humans with diabetes mellitus. In one study, elevated plasma CoP concentration was an independent risk factor for developing diabetes mellitus (140). Other studies found that elevated plasma CoP concentration was also a predictor of the development of chronic kidney disease during a 10-year follow-up in human patients with newly diagnosed type 2 diabetes (141, 142), and was associated with the risk of severe renal outcomes independent of usual covariates such as age, duration of diabetes mellitus, systemic blood pressure, and serum HbA1c concentration (143). The increase in plasma CoP concentration in patients with diabetes mellitus could be explained by chronic osmotic diuresis and decreased cellular concentration in V_2_ receptors, which subsequently leads to a blunted response to AVP in the renal distal tubule and collecting ducts (144, 145).

The most recent abstract (2024) reported the measurement of plasma AVP and serum CoP concentrations under hypo-osmolar (water load test), iso-osmolar (baseline) and hyper-osmolar (MWDT) conditions (77). In this study, both plasma AVP and serum CoP decreased after the water load test and increased after the MWDT. Correlation between plasma AVP and serum CoP was moderately positive (Pearson coefficient *r* = +0.52) (77).

## 7 Conclusion

This review provides an update on the physiology of AVP hormone and the limitations of its measurement in both human and veterinary medicine. Copeptin, a 39-amino acid glycopeptide comprising the C-terminal part of the AVP preprohormone and stoichiometrically secreted with AVP from the neurohypophysis in a 1:1 ratio, is a stable surrogate marker of AVP in humans. Additionally, baseline copeptin measurement coupled with CoP-based stimulation tests, such as hypertonic and arginine stimulation tests, offers excellent diagnostic accuracy in humans for diagnosing CDI, NDI and PP, especially in patients with complete forms of CDI and NDI. No AVP assay is commercially available in North America for veterinary clinical use, which likely explains the paucity of publications on water metabolism in the canine and feline species. Salivary and serum CoP have recently been successfully measured in dogs using research ELISA kits, and further research is highly encouraged in this field to validate and implement plasma CoP measurement for diagnosing PUPD disorders and as a biomarker of water balance and vascular tone.
